# The Internal Disintegration Effect in Microbial Aflatoxin Control Systems: From Detoxification to Suppression of Aflatoxin Biosynthesis in Toxigenic Fungi

**DOI:** 10.3390/toxins18090374

**Published:** 2026-08-30

**Authors:** Xue Zhang, Esa Abiso Godana, Kaili Wang, Hongyin Zhang, Qiya Yang

**Affiliations:** School of Food Science and Engineering, Jiangsu University, 301 Xuefu Road, Zhenjiang 212013, China; bbxzx1120@163.com (X.Z.); esaabiso@gmail.com (E.A.G.); 1000005483@ujs.edu.cn (K.W.)

**Keywords:** biodegradation, external pressure, internal disintegration

## Abstract

Aflatoxins (AFs) are highly carcinogenic mycotoxins produced by toxigenic fungi, posing serious threats to food safety, animal production, and human health. Microbial aflatoxin control has recently emerged as a promising, sustainable, and environmentally friendly strategy for reducing aflatoxin contamination. However, growing evidence suggests that reductions in AF levels within microbial treatment systems may arise not only from direct toxin degradation, but also from inhibition of fungal growth, adsorption or sequestration processes, and suppression of AF biosynthesis. In microbial co-culture systems involving viable toxigenic fungi, exogenous microorganisms and their metabolites can establish persistent ecological stress through nutrient competition, oxidative stress, and interspecies signaling. These stresses activate fungal cell wall integrity pathways, MAPK signaling cascades, and transcriptional regulatory networks, leading to membrane remodeling, alterations in lipid and energy metabolism, and redistribution of cellular resources. As a consequence, fungal physiology progressively shifts from a growth- and toxin-production-oriented state toward a survival- and defense-oriented state, resulting in impaired growth and reduced AF biosynthesis. This review proposes the internal disintegration framework, which conceptualizes AF suppression as a progressive loss of toxin-producing capacity caused by sustained microbial-induced physiological remodeling rather than solely by fungal growth inhibition or toxin degradation. It will provide new perspectives for developing precise, efficient, and eco-friendly dual-target control strategies.

## 1. Introduction

### 1.1. Aflatoxin Risk

Aflatoxins (AFs) are secondary metabolites produced primarily by *Aspergillus* genus fungi, mainly *Aspergillus flavus* and *Aspergillus parasiticus*, which contaminate grains, nuts, and feedstuffs [1,2,3]. Aflatoxin B1 (AFB1) is the most dangerous of all AFs and one of the most common and prevalent types found in food and feed [4]. It is significantly linked to the development of hepatocellular carcinoma and mainly causes liver damage [5]. Additionally, the International Agency for Research on Cancer has designated AFB1 as a Group 1 carcinogen due to its strong hepatotoxicity, immunotoxicity, mutagenicity, and carcinogenicity [4,6]. Since their widespread presence in a variety of foods and agricultural products, as well as the difficulty of detecting, eliminating, and controlling them, AF contamination continues to be a major risk factor in the sectors of public health, food safety, and feed safety worldwide [2,7,8].

### 1.2. Detection and Monitoring

Effective risk assessment and regulatory enforcement of AFs rely heavily on rapid, sensitive, and reliable analytical techniques. Traditional chromatographic methods such as high-performance liquid chromatography and liquid chromatography-tandem mass spectrometry, as well as immunoassays including enzyme-linked immunosorbent assay, have long served as reference standards due to their high accuracy and specificity [9]. However, these methods are often associated with laborious sample pretreatment, high operational cost, and limited suitability for rapid on-site screening, which restrict their application in large-scale agricultural monitoring [10]. It should be noted that detection strategies can be broadly divided into two categories: detection of toxigenic fungi and direct quantification of AFs. Molecular and microbiological methods are primarily used to identify toxigenic fungal species and assess contamination risk, whereas chromatographic, immunological, spectroscopic, and sensor-based methods are designed to quantify AF molecules directly. Because this review focuses on AF reduction mechanisms, only methods related to AF monitoring are briefly discussed below.

In recent years, spectroscopic techniques have emerged as powerful alternatives for non-destructive and rapid detection of AFs. Among them, near-infrared, Fourier transform near-infrared spectroscopy and Raman spectroscopy have been extensively investigated for the quantitative prediction of AFB1 in cereals [11,12,13,14,15] (Table 1). For instance, characteristic wavelength optimization combined with chemometric modeling significantly improves the predictive performance of NIR-based models for AFB1 detection in maize [11]. Similarly, Fourier transform NIR spectroscopy coupled with advanced feature selection algorithms has been shown to enhance model robustness and accuracy in corn AFB1 quantification [14]. These studies demonstrate that spectral preprocessing and wavelength selection are critical for overcoming the strong collinearity and high dimensionality of spectral datasets, enabling accurate, rapid, and non-destructive mycotoxin monitoring.

Apart from optical spectroscopy, sensing technology has also received increasing attention in recent years [16,17,18,19,20,21]. The integration of microwave sensing technology with deep learning frameworks achieves high prediction accuracy and demonstrates the feasibility of a real-time, non-destructive and intelligent mold toxin monitoring system [22]. Rather than serving as an independent detection technology, artificial intelligence functions primarily as a data-analysis tool. Machine learning and deep learning algorithms can improve feature extraction, spectral preprocessing, pattern recognition, and predictive modeling from complex NIR, Raman, and sensor datasets. Their major contribution lies in enhancing robustness and accuracy when handling high-dimensional and highly correlated data. Compared with traditional single-task models, these emerging technology improve data utilization efficiency and reduce model redundancy, highlighting the potential of artificial intelligence-driven sensing systems in agricultural safety evaluation [23,24,25]. In addition, immunochemical amplification strategies have pushed detection sensitivity to ultratrace levels [13].

In general, these advanced analytical techniques facilitate the systematic monitoring of AFs in rice, corn, nuts, spices and feed matrices, revealing the contamination levels and highlighting the urgency of taking effective control measures.

### 1.3. Prevention Strategies

The control strategies for AFs can generally be categorized into pre-contamination control and post-contamination remediation, which has gained broad acceptance in the current literature on food safety and mycotoxins [26]. Pre-contamination control focuses on using a variety of intervention strategies, including ecological regulation, agricultural management, and storage condition optimization, to prevent mycotoxin colonization and toxin biosynthesis before pollution happens. This effectively reduces the risk of AFs entering the food chain at the source [27]. Because AF contamination can occur throughout crop growth, harvesting, storage, and processing, pre-contamination control has the typical feature of “prevention throughout the entire process”, and is currently recognized as one of the most economically viable and sustainable control strategies. In contrast, post-contamination remediation targets commodities that have already been contaminated and includes several distinct approaches, such as adsorption/sequestration, physical detoxification, chemical detoxification, microbial transformation, and enzymatic degradation. These strategies differ in their mechanisms and targets, ranging from toxin removal or inactivation to biological conversion into less toxic products. Among them, microbial and enzymatic approaches have attracted increasing attention because of their environmental compatibility, high selectivity, and potential for sustainable AF control.

Environmental stress is thought to be the primary ecological element causing toxin-producing fungus infection and toxin manufacturing during the field stage. In addition to weakening the plant’s resistance, stress conditions like drought, high temperatures, pest damage, and mechanical injury can activate the AFs biosynthesis gene cluster by controlling the redox state and signaling pathways, greatly raising the risk of AF accumulation [3,27]. Thus, the likelihood of *Aspergillus* invasion can be successfully reduced by breeding stress-resistant cultivars, applying sensible irrigation and fertilization, minimizing insect damage, and improving agronomic techniques. Because of this, the use of hostile microbes in biological control has become increasingly important in recent years [28]. For instance, certain *Bacillus*, *Pseudomonas*, and yeast species can secrete antimicrobial metabolites or interfere with the regulatory network of toxin biosynthesis, achieving ecological suppression of toxigenic fungi [28,29].

In the post-harvest stage, storage environment management is another vital component of source control. Temperature and humidity directly determine the growth and toxin-producing capacity of *Aspergillus*. Measures such as rapid drying to reduce grain moisture content, maintaining low temperatures or controlled atmospheres, and improving warehouse ventilation can significantly inhibit fungal proliferation and toxin accumulation. Overall, by intervening in multiple stages of the survival and metabolism of toxigenic fungi, pre-contamination control strategies provide an economically effective and sustainable front-line guarantee against AF contamination.

In contrast, post-contamination remediation focuses on detoxifying agricultural commodities that have already been contaminated with AFs [7,30]. It serves as an important supplement to the pre-contamination control measures. In the actual production system, due to climate change, fluctuations in storage conditions, and the complexity of the supply chain, it is difficult to completely avoid AF contamination. Therefore, developing efficient and safe end-of-line detoxification technologies holds significant practical significance. In recent years, emerging physical detoxification technologies such as microwaves, infrared rays, and low-temperature plasma have shown excellent sterilization and toxin degradation effects in rice [17,31,32]. However, each of these technologies has its own limitations, such as uneven heating, insufficient penetration, or sensitivity to parameters, which have restricted their stable large-scale application. Microbial degradation of AFs has developed into a significant green detoxification strategy due to its environmental friendliness and potential high selectivity [33,34]. This progress is attributed to the continuous isolation and identification of microorganisms capable of degrading AFs and their key enzymes.

Early research primarily focused on screening non-pathogenic bacteria and fungi with AFB1-degrading capabilities, assessing their degradation efficiency, and identifying degradation products, thereby providing a feasible microbial resource base for detoxification technologies [33]. With the introduction of multi-omics technologies (transcriptomics, metabolomics, and proteomics), the microbial degradation mechanism has gradually shifted from “phenomenon description” to “mechanism analysis” [35,36,37]. These involve extracellular or intracellular oxidation, reduction, and hydrolysis reactions mediated by enzyme systems such as laccases, peroxidases, and lactonases. These reactions destroy the key structural components responsible for AFB1’s toxicity, converting it into less toxic metabolites [36,38,39]. From the perspective of research development, advances in this field can be categorized into two complementary directions. On the one hand, Ouyang et al. systematically reviewed aflatoxin-degrading microorganisms and their associated enzymatic systems, highlighting the central roles of enzymes such as laccases and peroxidases in AFB1 biotransformation and providing a foundation for the development of detoxification technologies [33]. On the other hand, as exemplified by the review of Ranjith Arimboor [39], the research perspective has further delved into the chemical mechanisms and safety aspects, indicating that the degradation process begins with enzymatic reactions such as oxidation and hydroxylation, generating intermediate products with lower chemical stability, which are then gradually transformed into low-molecular-weight substances. It also emphasizes the importance of conducting systematic toxicity evaluations of the complete degradation pathways and end products. These two types of research collectively demonstrate that microbial detoxification has evolved from early-stage efficiency screening to in-depth exploration of degradation mechanisms and safety.

### 1.4. Aim of This Review

**Figure 1:**
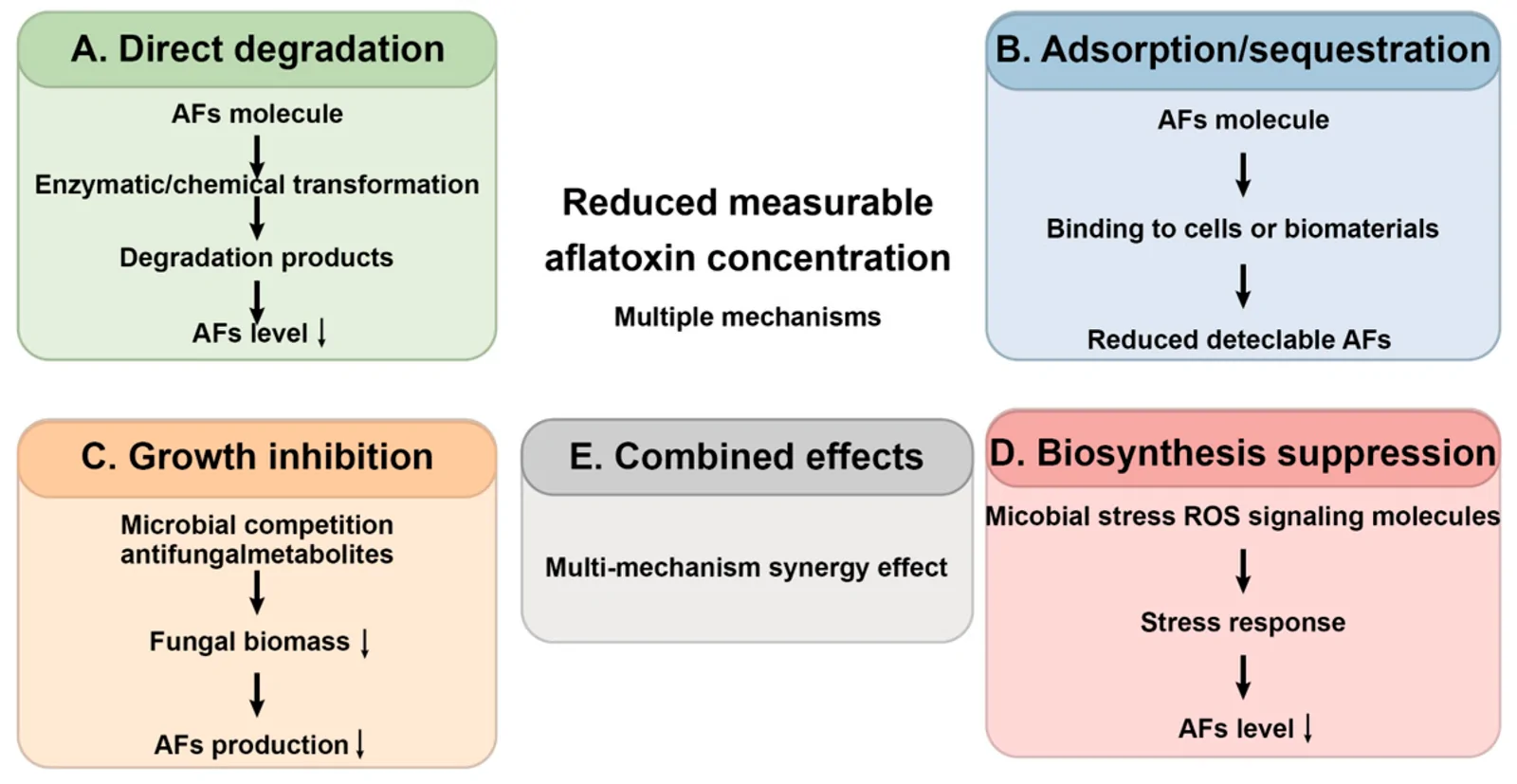
Mechanistic Classification of Processes Leading to Reduced Aflatoxin Levels. Note: The thick arrows in the figure indicate the result-oriented aspect, while the thin arrows represent the decrease in content.

Among the various AF control strategies described above, microbial control systems are unique because they may affect both AF molecules and the physiology of viable toxigenic fungi. The previous research largely remains within a unidirectional action framework of “exogenous microorganism-toxin molecule,” primarily understanding microbial AF-control system as a process of toxin removal or transformation (Figure 1), with insufficient attention paid to the physiological responses of the toxigenic fungi within this system [39,40]. In microbial co-culture systems containing viable toxigenic fungi, toxin reduction may be accompanied by fungal physiological responses. Such responses are not expected in purified enzyme or cell-free degradation systems. Recent studies suggest that toxigenic fungi are not passive participants in microbial degradation systems. During competition for nutrients and space, exogenous microorganisms release metabolites, signaling molecules, and reactive oxygen species (ROS). These factors impose sustained sublethal stress on fungal cells and trigger adaptive responses [33,41]. These reactions not only affect the growth and development of fungi, but also significantly interfere with the biosynthesis process of AFs by regulating the oxidative stress network, lipid metabolism and related signaling pathways of secondary metabolism [27,41].

An increasing number of studies have shown that ROS is not only a factor causing cell damage, but also an important signaling node regulating the stress response and toxin metabolism of fungi. Under continuous stress conditions, ROS can activate signaling pathways such as MAPK and cell wall integrity (CWI), triggering transcriptional regulatory networks and energy metabolism reconfiguration, causing fungi to gradually shift from a “growth–toxin production” state to a “survival–defense” state [27,42]. For instance, recent studies on *A. flavus* have revealed that oxidative stress significantly alters lipid composition, mitochondrial function, and energy metabolism levels, accompanied by a decrease in the synthesis ability of AFs [27]; at the same time, Cdc42 and RacA, among other Rho GTPases, participate in regulating ROS balance, cell polarity, and energy significant inhibition of AFs synthesis [42]. These results suggest that the continuous reductions in measured AF levels may arise from direct degradation, adsorption, growth inhibition, suppression of biosynthesis, or combinations of these processes.

**Figure 2:**
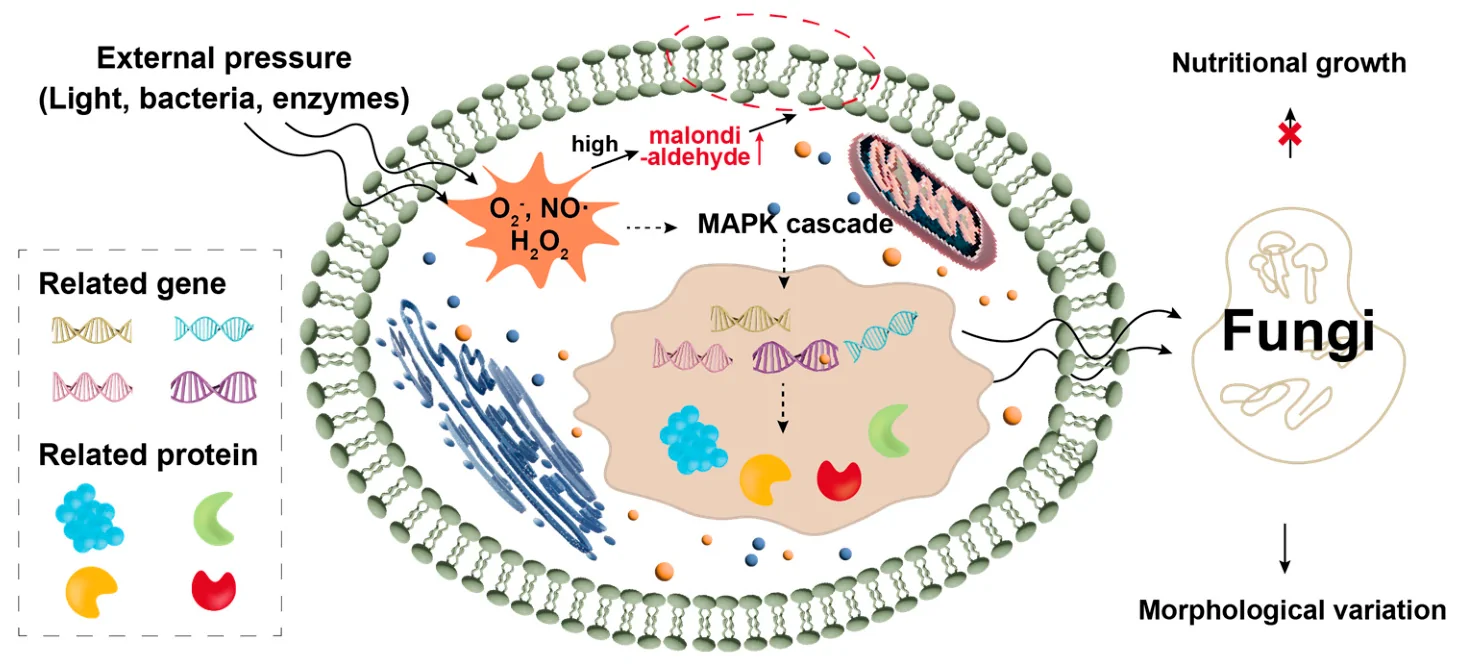
Proposed role of internal disintegration in microbial aflatoxin suppression. Note: The internal disintegration framework does not represent aflatoxin biodegradation per se. Instead, it describes a stress-mediated physiological process occurring in viable toxigenic fungi that may contribute to reduced aflatoxin accumulation through suppression of fungal growth and aflatoxin biosynthesis.

Conventional biodegradation-centered models, by overlooking the physiological feedback of toxigenic fungi and lacking understanding of the cumulative effects of long-term stress, struggle to explain the phenomenon of persistent toxin suppression rather than short-term decline observed in practice [40]. Therefore, it is necessary to broaden the conceptual framework of microbial aflatoxin control beyond direct biodegradation. While biodegradation refers specifically to the transformation of existing aflatoxin molecules, microbial control systems may additionally reduce aflatoxin accumulation through fungal growth inhibition, adsorption, and suppression of de novo aflatoxin biosynthesis. The proposed internal disintegration framework is intended to explain the latter processes rather than redefine biodegradation itself [33,43] (Table 2). Based on this, this paper, from the perspective of “internal disintegration”(Figure 2), focuses on exploring the mechanism by which the physiological state remodeling of toxigenic fungi contributes to AF suppression, aiming to provide a novel theoretical basis for optimizing biological control strategies against AFs. Relevant literature was searched in Web of Science, PubMed, Scopus, and Google Scholar, with the final search completed in May 2026. The search strategy combined terms related to aflatoxin contamination, Aspergillus flavus, microbial degradation, biological control, oxidative stress, reactive oxygen species, transcriptional regulation, lipid metabolism, and aflatoxin biosynthesis. Studies were selected based on their relevance to microbial aflatoxin-control systems and their contribution to understanding fungal physiological responses and toxin regulation. Priority was given to peer-reviewed articles published during the last five years (from 2021 to 2026); however, older publications were included when they constituted foundational studies, first descriptions of major regulatory pathways, landmark discoveries, or frequently cited reference work essential for interpreting current findings. It should be emphasized that the proposed “internal disintegration” model is applicable primarily to systems in which viable toxigenic fungi coexist with degrading microorganisms or their biologically active metabolites. In contrast, purified enzymes, cell-free extracts, or isolated catalytic systems may contribute to AF removal through direct degradation or transformation, but cannot induce physiological remodeling of toxigenic fungi in the absence of living fungal cells.

It should be noted that the conceptual framework proposed in this review is synthesized from evidence obtained at different experimental levels. Direct evidence from microbial AF-control systems demonstrates that microbial interactions can reduce AF accumulation and alter fungal physiology, whereas many of the downstream mechanisms discussed herein, including oxidative stress signaling, MAPK and cell wall integrity pathway activation, transcriptional reprogramming, lipid metabolic remodeling, and energy redistribution, are supported partly by studies conducted under abiotic stress conditions (e.g., temperature stress, ethanol treatment, pulsed light, and plasma-activated water), gene-deletion analyses, or investigations in Aspergillus species other than *A. flavus*. Therefore, the proposed internal disintegration framework should not be interpreted as a fully validated causal pathway demonstrated within a single experimental system. Rather, it represents a mechanistic synthesis integrating direct observations from microbial AF-control studies with broader knowledge from fungal stress biology. Throughout this review, direct evidence from microbial degradation or antagonistic systems is distinguished from mechanistic extrapolations derived from related physiological and molecular studies. Consequently, the framework should be regarded as a hypothesis-generating model that provides a coherent explanation for persistent AF suppression and offers experimentally testable directions for future validation.

### 1.5. Conceptual Definition of the Internal Disintegration Effect

The internal disintegration effect is defined as a progressive loss of toxin-producing capacity in viable toxigenic fungi subjected to persistent microbial-induced sublethal stress. Unlike conventional microbial antagonism, which primarily explains AF reduction through inhibition of fungal growth or biomass accumulation, the internal disintegration effect emphasizes coordinated physiological deterioration occurring prior to complete growth arrest (Table 3). This process involves sustained oxidative imbalance, transcriptional rewiring, metabolic resource reallocation, polarity disruption, and structural instability, ultimately leading to suppression of AF biosynthesis.

Importantly, although substantial evidence supports individual components of this framework, the complete sequence linking microbial pressure, oxidative imbalance, transcriptional reprogramming, metabolic remodeling, and long-term suppression of AF biosynthesis has not yet been experimentally verified within a single microbial–fungal interaction system and therefore remains a working hypothesis requiring further validation.

## 2. Perception of Coercion and Activation of Pathways

### 2.1. Stress Factor

In the microbial-mediated degradation system of AFs, exogenous stress is not a single pressure but a complex stress environment composed of multiple biological and physicochemical pressures. Firstly, the degrading microorganisms directly compete with the toxigenic fungi for nutrients, especially in the consumption of available carbon sources, nitrogen sources, and trace metal elements. The limitation of these resources can significantly alter the metabolism of the fungi [40]. For instance, *Bacillus subtilis* and *Pseudomonas putida* isolated from farmland soil can efficiently degrade AFB_1_, and significantly inhibit the growth and toxin production ability of *Aspergillus flavus* by competing carbon and nitrogen sources during growth [33].

Many reported degrading strains secrete organic acids, antibacterial secondary metabolites and volatile compounds during their growth, which can directly interfere with the integrity of the fungal cell membrane and intracellular homeostasis. For instance, lactic acid bacteria such as *Pediococcus pentosaceus* and *Weissella paramesenteroides* isolated from kimchi can produce metabolites such as lactic acid and 4-hydroxybenzaldehyde, significantly inhibiting the mycelial growth of *A. flavus* and disrupting its cell membrane integrity [50]. Additionally, volatile organic compounds such as dimethyl disulfide and dimethyl trisulfide produced by *Bacillus flexus* TR-1, can completely inhibit spore germination and toxin production of *A. flavus* [29]. Phenylacetaldehyde and 3,7-dimethyl-1-octanol produced by *Bacillus paramycoides* R2 also exhibit strong inhibited activity [51]. More importantly, the metabolic activities of degrading microorganisms often result in the formation of reactive oxygen species (ROS) or pro-oxidative environments, subjecting toxigenic fungi to long-term sub-lethal oxidative stress. For example, when lactic acid bacteria are co-cultured with *A. flavus*, their metabolites can induce the accumulation of intracellular ROS in the fungus, thereby triggering oxidative stress responses and weakening the metabolic activity of the fungus [51]. At the same time, during the catalytic oxidation and degradation of AFB_1_ by laccase, peroxidase and other degrading enzymes, the generation of ROS may also occur, further exacerbating the oxidative damage to the fungus [39].

In addition, recent studies have gradually focused on the role of cross-species signaling molecules. Quorum sensing molecules and extracellular signaling factors released by degrading microorganisms can be perceived by fungi, thereby triggering their stress response pathways. These signals do not aim to directly kill the fungi but rather continuously disrupt their physiological rhythms and metabolic balance, resulting in increased allocation of cellular resources toward stress-response pathways and reduced investment in growth-associated and secondary metabolic processes. For instance, when *Pediococcus pentosaceus* is co-cultured with *A. flavus*, the metabolites it secretes, such as 2’,3’-cAMP and adenine, may interfere with the fungal nucleoside metabolic pathway, inhibiting its energy metabolism and growth [50]. Therefore, in microbial AF control systems, exogenous microorganisms are not only “detoxification executors” but also “long-term stressors”, laying the foundation for subsequent internal physiological disintegration.

### 2.2. Stress Reaction

Oxidative stress is widely recognized as a central integrative hub linking external environmental pressures to the internal regulatory networks of fungi. In diverse microbial antagonism systems or biodegradation processes, pathogenic fungi are frequently exposed to stressors that disrupt cellular redox homeostasis, leading to a significant accumulation of ROS, particularly hydrogen peroxide (H_2_O_2_) and superoxide anions (O_2_^−^). These ROS not only cause oxidative damage to lipids, proteins, and nucleic acids, but also function as signaling molecules that modulate gene expression and metabolic pathways. To mitigate oxidative damage, fungi have evolved a range of antioxidant defense mechanisms, including the induction of enzymes such as superoxide dismutase (SOD), catalase (CAT), and components of the glutathione and thioredoxin systems [52]. However, under prolonged or intense exogenous stress, these defense systems are often insufficient to fully neutralize ROS accumulation, resulting in sustained oxidative pressure within the cell.

Several transcriptomic studies conducted under specific oxidative-stress conditions have reported coordinated changes in gene expression in *A. flavus*, including upregulation of antioxidant-defense pathways and reduced expression of certain aflatoxin-biosynthetic genes. Specifically, in some experimental systems, genes associated with antioxidant defense, cellular repair, and stress adaptation were upregulated, whereas reduced expression of aflatoxin-biosynthetic genes was observed [53,54]. However, these responses were not consistently reported across all oxidative-stress conditions. This opposing expression pattern indicates that oxidative stress acts not merely as a damaging factor, but as a critical signaling node that actively regulates the allocation of metabolic resources. Under certain prolonged stress conditions, fungal cells may reprioritize energy utilization, diverting resources from secondary metabolism toward cellular maintenance and stress adaptation, such as AFs, toward essential processes required for survival, maintenance, and repair. Nevertheless, the extent of this metabolic reallocation appears to vary among experimental systems.

Furthermore, studies suggest that redox-sensitive transcription factors, such as AtfB and Yap1 homologs, play pivotal roles in mediating this regulatory shift by sensing intracellular ROS levels and modulating downstream gene expression networks [55]. Consequently, prolonged oxidative stress has been associated with reduced aflatoxin biosynthesis and broader metabolic changes in several experimental studies. However, these observations should be interpreted within the specific biological and environmental contexts in which they were obtained. This adaptive strategy provides a robust physiological explanation for the observed reduction in toxin production under continuous environmental stress, highlighting the dual role of oxidative stress as both a threat and a regulatory signal in fungal biology.

Importantly, the effects of oxidative stress on aflatoxin biosynthesis are highly context-dependent and may vary according to stress intensity, exposure duration, developmental stage, fungal strain, and environmental conditions. Moderate oxidative stress has been reported to stimulate aflatoxin production in some systems, whereas severe or prolonged stress may suppress fungal growth and secondary metabolism. Therefore, oxidative stress should not be interpreted as a universal negative regulator of aflatoxin biosynthesis, but rather as a dynamic signal whose effects depend on the physiological context.

### 2.3. Activation of Pathways

**Figure 3:**
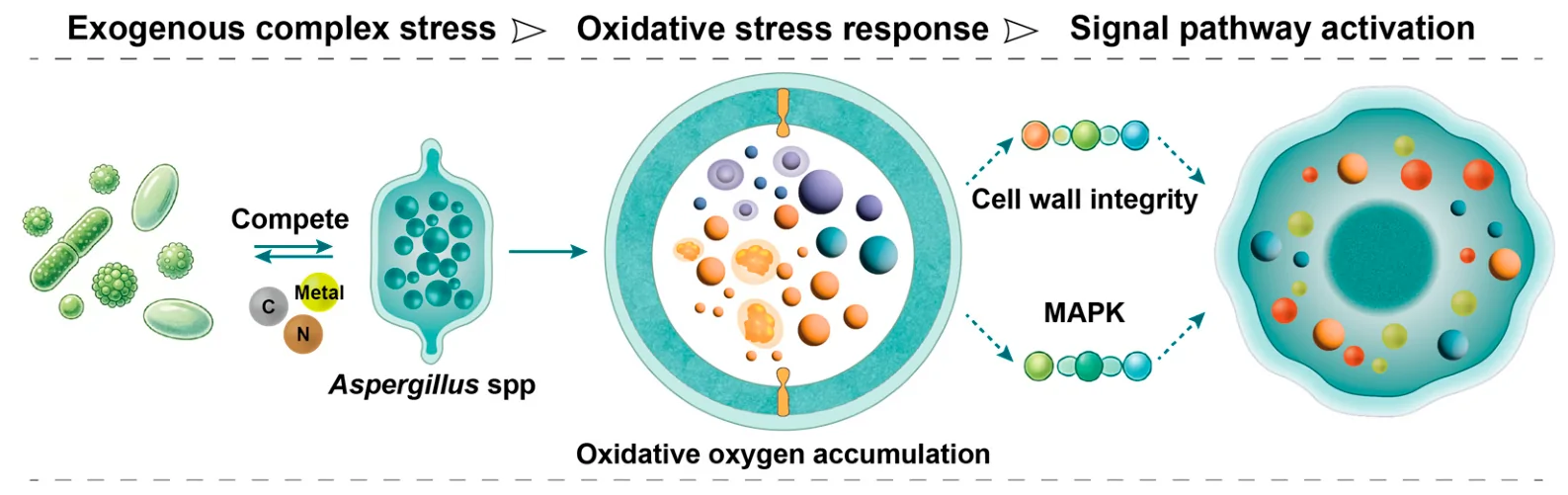
Stress-mediated suppression of aflatoxin biosynthesis in microbial–fungal interaction systems. Note: Dashed connections represent mechanistic interpretations synthesized from independent studies rather than direct experimental validation.

In fungal cells, ROS simultaneously perform the roles of damage factors and signaling molecules. When the ROS level rises and causes structural abnormalities in the cell wall or membrane system, fungi rapidly activate multiple stress signaling pathways through membrane receptors. Among the cell wall integrity (CWI) pathway and the MAPK cascade reaction are the most crucial (Figure 3). The CWI pathway can sense changes in cell wall stress and transmit signals to the nucleus through the downstream protein kinase network, thereby inducing the expression of genes related to cell wall repair, membrane lipid remodeling, and antioxidant defense. For example, studies have shown that various volatile organic compounds (such as 1-pentanal and 1-heptanol) can induce cell membrane damage and lipid peroxidation of *A. flavus*, thereby activating the MAPK signaling pathway and significantly upregulating the expression of genes related to cell wall remodeling and oxidative stress response [51]. Additionally, when *A. flavus* is exposed to oxidative stress conditions, the expression of MAPK signaling pathway-related genes in the fungus undergoes significant changes, suggesting that this pathway plays a key role in sensing and transmitting oxidative stress signals [56].

It is worth noting that recent studies have revealed a significant bidirectional regulatory relationship between the CWI pathway and the ROS generation system. For instance, small GTPases and their downstream effectors can influence the level of ROS production by regulating the NADPH oxidase complex, while ROS can continuously activate the CWI signal. This positive feedback mechanism helps fungi maintain structural integrity in the short term, but under long-term exogenous stress conditions, it will instead exacerbate energy consumption and metabolic burden, ultimately leading to failure in repair and structural instability. For example, the transcription factor AtfA in *A. flavus*, as a downstream response factor of the MAPK pathway, not only regulates the expression of antioxidant enzyme genes to cope with oxidative stress, but also participates in the regulation of fungal development and toxin synthesis; when AtfA is absent, the sensitivity of the strain to oxidative stress significantly increases, and the growth and spore production of the mycelium are both inhibited. Similarly, the epigenetic regulatory factor SntB participates in the oxidative stress response of *A. flavus* by regulating the expression of the catalase gene *catC*. Its absence leads to an increase in intracellular ROS levels, and the growth of mycelium and toxin synthesis are significantly decreased [57]. This process provides an important signaling basis for subsequent metabolic reconfiguration and morphological abnormalities.

## 3. Dynamic Regulation of Transcription Factors

**Figure 4:**
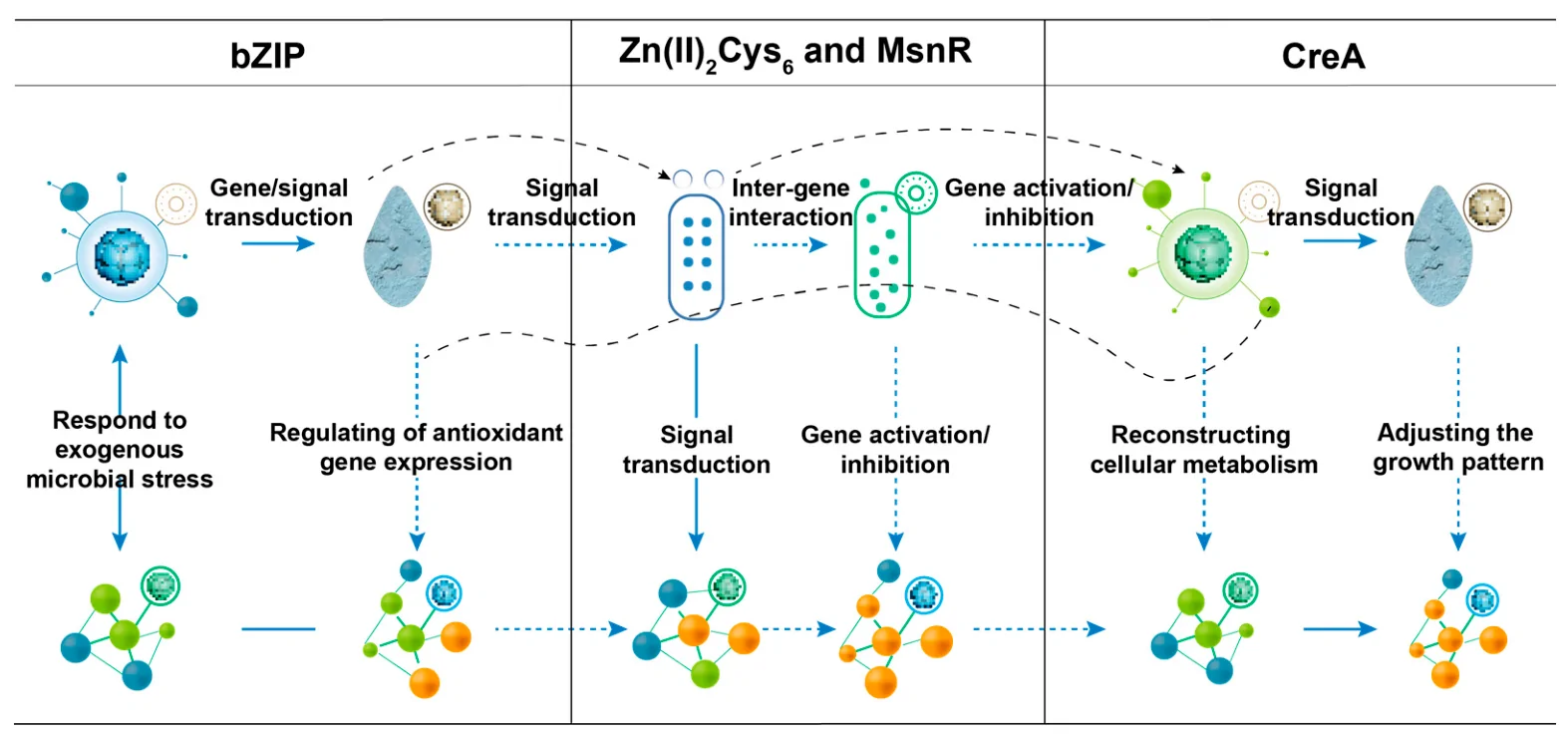
The dynamic regulation process of aflatoxin transcription factors. Dashed line pathways shown in this figure are inferred from broader fungal stress biology and have not yet been directly demonstrated in microbial–toxigenic fungal interaction systems.

Within the persistent stress environment established by exogenous microorganisms, toxigenic fungi undergo extensive reprogramming of gene expression profiles. This process is not merely a passive adaptive response but involves coordinated regulation by a highly conserved and interconnected transcriptional regulatory network. As central regulatory nodes, transcription factors perceive and integrate diverse environmental and intracellular signals, translating ecological pressures into adjustments of metabolic, developmental, and stress-response pathways through complex synergistic and antagonistic interactions. In Aspergillus flavus, the activities of key transcription factors under stress are highly dynamic and context-dependent, varying with stress intensity, exposure duration, developmental stage, and physiological status. These regulators can modulate secondary metabolism, including AF biosynthesis, either positively or negatively depending on the prevailing cellular conditions. Therefore, elucidating the behavior of stress-responsive transcription factors is essential for understanding how the proposed “internal disintegration” effect is transmitted from signal perception to downstream metabolic regulation (Figure 4).

### 3.1. bZIP

bZIP-type transcription factors play a central role in the stress response of eukaryotes. The activities of homologous proteins in *A. flavus*, such as AtfA and AtfB, are directly regulated by the cellular redox state. When exogenous microorganisms cause an increase in intracellular ROS levels, specific cysteine residues undergo reversible oxidation, altering the conformation, subcellular localization, or protein stability of the transcription factors, thereby activating their transcriptional activity. These activated bZIP factors preferentially bind to the promoter regions containing specific antioxidant response elements (ARE), strongly driving the expression of a series of antioxidant enzymes (such as catalase Cat1, superoxide dismutase Sod1) and thioredoxin system genes, in an attempt to restore redox homeostasis. For instance, studies have shown that transcription factors in *A. flavus*, AtfA and AtfB, directly regulate the expression of the catalase gene (*cat1*) and superoxide dismutase gene by binding to the CRE elements in the promoter regions of the target genes, thereby responding to oxidative stress [58]. Additionally, the deletion of AtfA significantly reduces the growth rate and sporulation ability of *A. flavus* under oxidative stress and leads to enhanced sensitivity to H_2_O_2_, confirming its core role in antioxidant defense [53]. Transcriptomic analysis also reveals that the expression of multiple bZIP transcription factors in *A. flavus* undergoes significant changes under temperature or oxidative stress conditions, accompanied by differential expression of antioxidant enzyme genes [56].

However, the consequences of stress-responsive transcriptional activation extend beyond antioxidant defense and include broader changes in metabolic resource allocation and secondary metabolism. Studies have shown that bZIP factors, as global metabolic regulators, can inhibit gene clusters related to secondary metabolism. For instance, AtfA has been reported to influence the expression of the key regulatory genes *AflR* and *AflS*, which coordinate the activation of the AF biosynthetic cluster. However, the direction and magnitude of this regulation appear to vary among experimental conditions, suggesting that AtfA functions within a broader stress-response network rather than acting as a simple repressor of AF biosynthesis. Through interactions with promoter regulatory elements and chromatin-remodeling factors, bZIP transcription factors may influence the accessibility and transcriptional activity of AF biosynthetic genes. These effects are likely integrated with oxidative stress signaling and broader metabolic regulatory networks. For example, a systematic knockout study of 15 bZIP transcription factors in *A. flavus* revealed that more than two-thirds of the bZIP members were involved in the regulation of AFs synthesis, and the deletion of *AtfA* and *AtfB* led to a significant decrease in AFB_1_ production, accompanied by altered expression of *AflR* and *AflS*. These findings suggest that AtfA and AtfB contribute to the coordination of oxidative stress adaptation and secondary metabolism. Importantly, their regulatory effects on AF biosynthesis appear to be context dependent and may vary according to oxidative stress intensity, developmental stage, and physiological conditions, indicating that bZIP factors play a crucial hub role in coordinating the oxidative stress response and secondary metabolism (Table 4) [59]. Similarly, CpcA (a bZIP transcription factor) not only participates in the amino acid starvation response, but its deletion also leads to a significant reduction in AFs synthesis, further confirming the function of bZIP factors in metabolic reprogrammin (Table 4) [59]. Moreover, MetR, as a bZIP factor involved in sulfur metabolism regulation, its deletion not only leads to a methionine nutritional defect but also completely blocks AFs synthesis, indicating that bZIP factors can indirectly affect toxin production through multiple metabolic pathways (Table 4) [59]. Therefore, under oxidative stress, bZIP transcription factors play an important role in reallocating cellular resources between stress adaptation, growth, and secondary metabolism. The balance among these processes is dynamic and depends on the intensity and duration of stress as well as the physiological state of the fungus. Consequently, oxidative stress may either promote or suppress AF biosynthesis under different conditions.

### 3.2. Zn(II)_2_Cys_6_ and MsnR

In the regulatory network of AFs biosynthesis, the Zn(II)_2_Cys_6_ type transcription factors play a central role. These factors are widely involved in the secondary metabolism, carbon and nitrogen source utilization, and growth and development of fungi. Among them, the pathway-specific regulatory factor AflR is a typical representative of this family [60]. Studies have shown that AflR not only directly activates the transcription of at least 17 genes in the AFs biosynthesis gene cluster, but its mutant lacking (Δ*AflR*) produces only one-thousandth of AFB_1_ on YES medium compared to the wild type, accompanied by sporulation defects and severe inhibition of sclerotium formation, indicating that AflR has multiple functions in coordinating toxin synthesis and development [60]. Under conditions of exogenous microbial stress, the expression of AflR is suppressed, and its function may also be inhibited. For example, the stress signal may change the nuclear localization or DNA binding ability of AflR through kinase-mediated phosphorylation modification. The activity changes of other global Zn(II)_2_Cys_6_ factors involved in carbon and nitrogen source utilization (such as FacB involved in acetate metabolism) will profoundly affect the size and flow of the acetyl-CoA pool, and acetyl-CoA is the starting unit of AFs polyketide compounds. When these global factors sense carbon source competition or metabolic stress, they will guide the carbon flow to central metabolic pathways such as the tricarboxylic acid cycle to maintain energy supply, thereby limiting AFs synthesis at the substrate level. For example, the newly identified Zn(II)_2_Cys_6_ transcription factor AfEcm22 (AFLA_008550) has been confirmed to affect acetyl-CoA synthesis by regulating the expression of genes related to the pyruvate dehydrogenase (PDH) complex; after knocking out AfEcm22, PDH activity decreases, acetyl-CoA content reduces, although the transcription level of the AFs gene cluster is upregulated, the AFB_1_ production is still significantly reduced [61]. This discovery reveals a new mechanism by which Zn(II)_2_Cys_6_ family factors indirectly regulate secondary metabolism by reshaping the primary metabolic flow.

The Msn family transcription factors (such as MsnA) are universal mediators for fungi to cope with various environmental stresses (such as oxidation, osmotic pressure, and nutrient stress). They are usually in an inhibitory state and are rapidly activated and enter the nucleus under stress conditions. The activation of Msn factors marks a physiological transition from growth-associated processes toward stress adaptation responses. The regulatory network of these factors extensively involves the accumulation of compatible solutes such as glycogen and glycerol, the upregulation of heat shock proteins, and the delay of the cell cycle process. In this mode, energetically demanding biosynthetic processes not essential for immediate stress adaptation, including the synthesis of AFs, are systematically suppressed. For example, after knocking out MsnA in *A. flavus*, colony growth is inhibited, but the spore production, AFs, and curacin production are significantly increased, while the intracellular ROS level is elevated, indicating that MsnA inhibits toxin synthesis and maintains redox homeostasis under normal circumstances [62]. Further studies have found that ethanol treatment can enhance the transcription of antioxidant enzyme genes (*CATA*, *SOD1*, etc.) by upregulating the expression of MsnA and bZIP transcription factor AP-1, and C_2_H_2_ factor MtfA, thereby inhibiting the synthesis of AFs; while the deletion of MsnA weakens the inhibitory effect of ethanol on toxin production [54]. This suppression is not achieved by directly targeting the Afl gene cluster, but by reshaping the physiological state of the entire cell and the priority of resource allocation, reflecting the profound connotation of “internal disintegration” at the systems biology level.

### 3.3. Carbon Metabolism (CreA)

In the ecological context of microbial AF-control system, the vigorous metabolism of exogenous degrading microorganisms rapidly consumes simple sugars in the environment, creating a “carbon source scarcity” phenomenon. This change is perceived by the membrane sensor system of *A. flavus*, leading to the de-repression or transformation of the carbon decomposition metabolite repression (CCR) mechanism mediated by CreA. Studies have shown that the wild-type strain of *A. flavus* grows vigorously and produces toxins normally on glucose-rich media, while the CreA gene knockout mutant (Δ*CreA*) exhibits significant phenotypic defects: regardless of the type of carbon source, the growth rate of the Δ*CreA* mycelium slows down, aerial hyphae decrease, conidial production significantly declines, and cell hydrophobicity is lost. This indicates that CreA is a key factor maintaining the normal growth and development of the fungus (Table 5) [63].

The activated CreA inhibits a series of genes encoding enzymes for breaking down complex carbon sources (such as starch and cellulose), which is an energy-saving strategy. For instance, the α-amylase activity of the Δ*CreA* strain was significantly higher than that of the wild type in a starch medium, and it was not inhibited by easily accessible carbon sources such as glucose, confirming that CreA represses the expression of hydrolytic enzyme genes at the transcriptional level (Table 5) [63]. More importantly, the synthesis of secondary metabolites is often associated with low growth rates and the utilization of special carbon sources. Studies have shown that CreA can directly bind to the promoter regions of some secondary metabolic gene clusters and inhibit their transcription. For example, in *A. flavus*, the deletion of CreA led to a significant increase in sclerotium production, which is the dormant structure of the fungus under adverse conditions, and its formation is closely related to the reduced carbon source utilization efficiency; at the same time, the AFB_1_ production of the ΔCreA strain was reduced to less than 1/16 of that of the wild type, and the expression of multiple structural genes (such as *AflD*, *AflM*, *AflO*, *AflP*) and the key regulatory gene *AflR* in the toxin synthesis gene cluster was significantly downregulated (Table 5) [63]. Further research revealed that the co-suppressor factors SsnF and RcoA in the CCR pathway interact with CreA to jointly regulate the synthesis of AFs; after knocking out *SsnF* or *RcoA*, the expression of AflR and AflS decreased, and the AFB_1_ production was reduced to 1/3 and 1/7 of that of the wild type, respectively, and the formation of sclerotia was completely blocked, indicating that the SsnF-RcoA complex, as a co-factor of CreA, plays a key role in carbon source signal transduction and toxin synthesis regulation [64].

The activated CreA inhibits a series of genes encoding enzymes for breaking down complex carbon sources (such as starch and cellulose), which is an energy-saving strategy. For instance, the α-amylase activity of the Δ*CreA* strain was significantly higher than that of the wild type in a starch medium, and it was not inhibited by easily accessible carbon sources such as glucose, confirming that CreA represses the expression of hydrolytic enzyme genes at the transcriptional level (Table 5) [63]. More importantly, the synthesis of secondary metabolites is often associated with low growth rates and the utilization of special carbon sources. Studies have shown that CreA can directly bind to the promoter regions of some secondary metabolic gene clusters and inhibit their transcription. For example, in *A. flavus*, the deletion of CreA led to a significant increase in sclerotium production, which is the dormant structure of the fungus under adverse conditions, and its formation is closely related to the reduced carbon source utilization efficiency; at the same time, the AFB_1_ production of the ΔCreA strain was reduced to less than 1/16 of that of the wild type, and the expression of multiple structural genes (such as *AflD*, *AflM*, *AflO*, *AflP*) and the key regulatory gene *AflR* in the toxin synthesis gene cluster was significantly downregulated (Table 5) [63]. Further research revealed that the co-suppressor factors SsnF and RcoA in the CCR pathway interact with CreA to jointly regulate the synthesis of AFs; after knocking out *SsnF* or *RcoA*, the expression of AflR and AflS decreased, and the AFB_1_ production was reduced to 1/3 and 1/7 of that of the wild type, respectively, and the formation of sclerotia was completely blocked, indicating that the SsnF-RcoA complex, as a co-factor of CreA, plays a key role in carbon source signal transduction and toxin synthesis regulation [64].

For AFs, the shift in carbon source type (from easily accessible sugars to lipids or amino acids) and the overall reduction in carbon flux, send a strong “shut down” signal to the secondary metabolic network through the transmission of factors such as CreA. Additionally, other transcription factors involved in carbon metabolism regulation also participate in this process. For instance, the deletion of the Zn_2_Cys_6_ type transcription factor AfEcm22 leads to a decrease in pyruvate dehydrogenase activity and a reduction in the acetyl-CoA pool. Although the transcriptional level of the toxin gene cluster is upregulated, the production of AFB_1_ still significantly decreases, indicating that CreA and its co-factors reshape the primary metabolic flow and limit toxin synthesis at the substrate level [61]. At the same time, epigenetic regulation also participates in this process: the treatment with leucine through inhibition of M^6^A methylation leads to a decrease in the mRNA stability of genes related to toxin synthesis (such as *AflC*, *AflD*, *AflK*), thereby inhibiting the synthesis of AFB_1_ [65]. Therefore, CreA is a key signal converter that transforms the nutritional competitive pressure from the external ecological niche into the reconfiguration of the internal metabolic network, ultimately leading to the “shutdown” of toxin synthesis.

## 4. Remodeling of Lipid Metabolism

**Figure 5:**
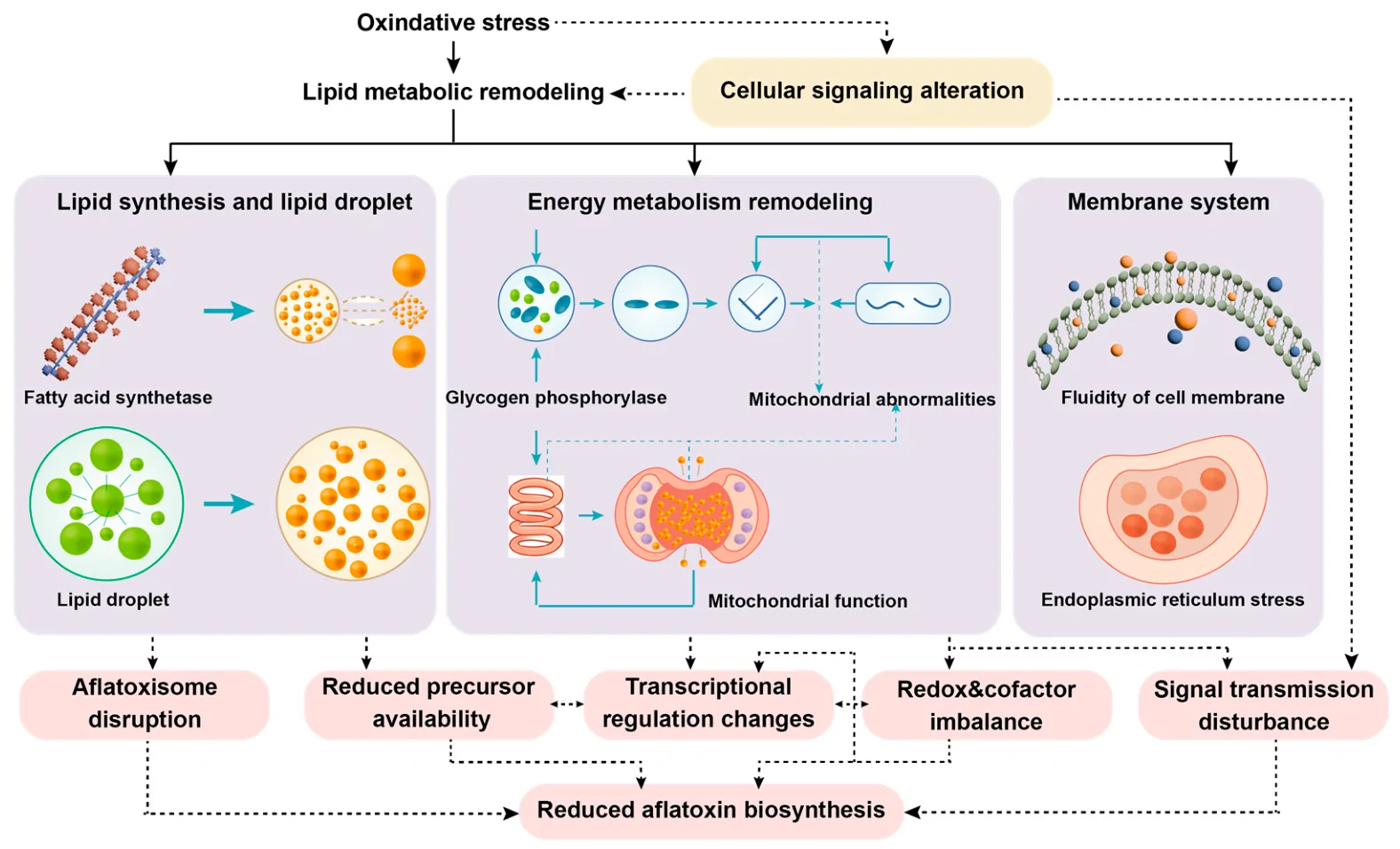
Proposed relationship between stress-induced lipid metabolic remodeling and suppression of aflatoxin biosynthesis in toxigenic fungi. Note: Persistent microbial-derived stress may induce lipid metabolic remodeling, including changes in fatty-acid composition, lipid droplet homeostasis, energy metabolism, membrane organization, and cellular signaling. These processes are associated with reduced aflatoxin production under specific experimental conditions. Solid arrows indicate relationships supported by experimental evidence, whereas dashed arrows indicate inferred mechanistic links that constitute the proposed Internal Disintegration Framework. The complete pathway should therefore be regarded as a working hypothesis rather than a fully validated causal sequence.

Lipid metabolism serves as a central hub of fungal life activities, providing precursors and platforms for membrane construction, energy storage, signal transduction, and secondary metabolism. Within the proposed internal disintegration framework, stress-induced lipid metabolic remodeling is hypothesized to contribute to the suppression of aflatoxin biosynthesis through changes in membrane organization, precursor allocation, and cellular signaling. While several components of this process have been experimentally demonstrated, the complete causal pathway remains inferential and requires further validation (Figure 5). This reorganization fundamentally undermines the material, energetic, and spatial foundations upon which AF biosynthesis relies, serving as a prerequisite for the collapse of its synthetic capacity.

### 4.1. Lipid Synthesis

AFs are polyketide secondary metabolites whose biosynthesis is initiated by the condensation of acetyl-CoA and malonyl-CoA, linking AF production to cellular carbon metabolism and lipid-associated metabolic pathways. Previous studies have shown that environmental stress can alter the expression of genes involved in fatty acid metabolism and lipid homeostasis, potentially affecting the metabolic context in which AF biosynthesis occurs. Lipidomic analyses revealed that *A. flavus* cultured at 28 °C, a condition permissive for AFB_1_ production, contained higher proportions of polyunsaturated fatty acids (e.g., C18:2 and C18:3) and a greater abundance of triglyceride species with unsaturated acyl chains than cultures grown at 37 °C, where AFB_1_ production was substantially reduced. These temperature-dependent changes in lipid composition were accompanied by differences in AFB_1_ accumulation, suggesting a potential association between lipid unsaturation status and aflatoxin biosynthesis (Table 6) [27]. Transcriptomic analyses further demonstrated that exposure to 37 °C was associated with altered expression of genes involved in fatty acid metabolism, indicating that temperature stress may influence AF production through broader lipid metabolic remodeling [27]. Similarly, deletion of the carbon catabolite repression factor CreA not only impaired mycelial growth and sporulation but also altered the expression of genes involved in carbon utilization and lipid metabolism, accompanied by reduced AF production [63]. Collectively, these observations support a correlation between lipid metabolic state and aflatoxin biosynthesis. However, the causal relationship between lipid remodeling and AF suppression has not yet been directly established and requires further experimental verification.

Lipid droplets, as cellular organelles for the storage of neutral lipids (mainly triglycerides and sterol esters), play a role as a “metabolic factory” in fungal secondary metabolism. There is evidence suggesting that certain enzyme complexes involved in AFs synthesis may be anchored on the surface of lipid droplets to take advantage of the enriched lipid precursors present there. For instance, the lipid droplet-related protein caleosin (AfPXG) has been proven to have peroxidase activity, and its absence leads to the inhibition of AFs mycelial growth, complete suppression of sporulation, and a reduction in AFB_1_ production by over 92%; while overexpression of AfPXG significantly increases the number and stability of lipid droplets, and simultaneously upregulates the expression of toxin synthesis gene clusters, increasing the secretion rate of AFB_1_ from 0.71 to 0.98 [66]. Under stress conditions, the biogenesis of lipid droplets is inhibited: the expression of core proteins decreases, the number of lipid droplets reduces, and their volumes shrink. For example, under heat stress (37 °C), the abundance of various lipid classes such as triglycerides in the mycelium significantly decreases, and lipid droplet accumulation decreases [27]. More importantly, the turnover (balance between synthesis and degradation) of lipid droplets is disrupted. Autophagy (especially lipophagy) may be activated to break down lipid droplets for energy supply, but this further depletes the reserve of raw materials for AFs synthesis. For instance, the global regulatory factor MsnA’s absence leads to inhibited colony growth, but the sporulation yield, AFs, and ergosterol production are significantly increased, while intracellular reactive oxygen levels increase, indicating that MsnA maintains redox homeostasis and inhibits toxin synthesis under normal conditions; and the regulation of MsnA may be partially achieved by influencing lipid metabolism and energy flow [62]. Therefore, the dynamic imbalance of the lipid droplet system destroys the subcellular platform for efficient toxin synthesis from both the “spatial organization” and “storage of raw materials” perspectives.

### 4.2. Energy Metabolism

Mitochondria are the energy factories of cells and also an important site for ROS production and stress sensing. Persistent ROS attacks triggered by exogenous microbial stress can damage the inner mitochondrial membrane, leading to reduced activity of the electron transport chain complex, leakage of proton gradient, and thus uncoupling of oxidative phosphorylation, resulting in a significant decrease in ATP synthesis efficiency. This reduction in energy availability induces metabolic adjustments that regulate ATP-consuming and ATP-generating pathways. For example, after pulsed light (PL) treatment of Aspergillus, the ATP content and ATPase activity in the mycelium significantly decreased, mitochondrial function was impaired, and energy metabolism was blocked [67]. Similarly, plasma-activated water (PAW) treatment also caused depolarization of mitochondrial membrane potential, decreased ATP levels, and ultimately led to the inactivation of Aspergillus [68]. The inhibition of the TOR signaling pathway by rapamycin also significantly affects energy metabolism, resulting in inhibition of mycelial growth and toxin synthesis, accompanied by lipid droplet accumulation, indicating that the energy redistribution process was activated [69]. Although pulsed light and plasma-activated water are not microbial control systems, these studies demonstrate that persistent oxidative stress can disrupt mitochondrial function and cellular energy metabolism in Aspergillus species. Therefore, they provide mechanistic clues regarding how similar stress responses might operate in microbial AF-control systems.

The homolog of AMP-activated protein kinase (AMPK), Snf1, plays the role of an energy sensor in fungi. When the ATP/AMP ratio decreases, Snf1 is activated, which then phosphorylates and regulates a series of downstream targets. The overall effect is to suppress energy-demanding anabolic processes (such as fat production and protein synthesis) and activate catabolic processes (such as fatty acid β-oxidation and autophagy) to regenerate ATP. Studies have shown that the Fus3-MAPK signaling pathway participates in energy metabolism regulation by interacting with the β subunit Gal83 of the Snf1/AMPK complex: Fus3 directly phosphorylates Gal83, thereby affecting the synthesis levels of acetyl-CoA and malonyl-CoA, and thus regulating the biosynthesis of AFs. The transcriptional level of the toxin gene cluster itself did not change significantly [70]. Under this global energy rearrangement scheme, AFs, as energy-intensive secondary metabolites with substantial requirements for carbon precursors and reducing equivalents, may be particularly sensitive to metabolic constraints under energy-limited conditions. This inhibition is systematic: not only the anabolic pathways themselves are inhibited, but the intensity of the auxiliary metabolic flows that provide NADPH, such as the pentose phosphate pathway, may also weaken. For example, the expression of the key enzyme 6-phosphogluconate dehydrogenase in the pentose phosphate pathway in the gal83 deletion strain is significantly downregulated, indicating that the reducing power supply is weakened [70]. Therefore, the inhibition of toxin synthesis may result from energy limitation and metabolic reallocation under conditions of reduced ATP availability, which redirects cellular resources toward essential maintenance processes.

In *A. flavus*, Rho GTPases Cdc42 and RacA play significant roles in energy metabolism regulation. The study found that in the Δcdc42 mutant, genes related to fatty acid β-oxidation and pyruvate metabolism were significantly downregulated, while in the Δ*racA* mutant, these pathways were upregulated, showing an opposite energy metabolism regulation pattern. Correspondingly, the content of acetyl-CoA increased in the Δcdc42 mutant, and lipid droplet accumulation significantly increased, while in the Δ*racA* mutant, the content of acetyl-CoA decreased, and lipid droplets decreased. Nevertheless, the mitochondrial membrane potential of both mutants increased, and ATP content was significantly higher than that of the wild type, indicating an enhanced energy metabolism rate [42]. This result reveals the functional differentiation of Cdc42 and RacA in regulating energy metabolism: Cdc42 may negatively regulate fatty acid breakdown and energy release, while RacA positively promotes these processes, and together they maintain the energy balance of Aspergillus flavus during growth, development, and toxin synthesis.

### 4.3. Membrane System Characteristics

The remodeling of membrane lipid composition (such as changes in the ratio of phosphatidylcholine to phosphatidylethanolamine, and variations in sterol content) directly affects the physical properties of the membrane, including fluidity, phase transition temperature, and the embedding environment of membrane proteins [71,72]. Studies have shown that the lipid composition of fungal membranes (especially the ratio of PC/PE and sterol content) is closely related to membrane fluidity and the function of membrane proteins, thereby influencing cellular metabolism and adaptability [72,73]. Under stress conditions, the unsaturation of membrane lipids often undergoes adjustment to reduce lipid peroxidation sensitivity, but this adaptive change is usually accompanied by an increase in membrane rigidity, thereby inhibiting the conformational dynamics and functional activity of membrane proteins (such as nutrient transport proteins and signal receptors) [27]. At the same time, lipid peroxides produced by oxidative stress (such as malondialdehyde, MDA) can directly damage the lipid bilayer structure and lead to a decrease in membrane integrity [74,75].

The degradation of this membrane system’s function will lead to multi-dimensional metabolic consequences. Firstly, the decrease in membrane fluidity will limit the efficiency of nutrient transmembrane transport, thereby reducing the uptake capacity of substrates such as glucose and amino acids, exacerbating the “nutrient deficiency” state within the cell [27]. Secondly, the damage to the membrane structure will weaken the maintenance ability of transmembrane ion gradients (such as H^+^ and Ca^2+^), thereby disrupting the intracellular ion homeostasis and energy metabolism processes, and affecting the excretion process of secondary metabolites, resulting in abnormal accumulation of toxin intermediates within the cell and generating feedback inhibition [76].

More importantly, many stress signaling pathways (such as the cell wall integrity pathway, CWI) have membrane-anchored receptors (e.g., Wsc family sensors) that rely on a stable membrane microenvironment to achieve precise signal perception and transmission [77,78]. Studies have shown that the CWI signaling pathway activates the Rho1-MAPK cascade reaction through the Wsc sensor and is highly sensitive to changes in membrane lipid composition. Membrane structural disturbances can significantly interfere with the efficiency of signal transmission [79,80]. Therefore, changes in membrane properties introduce “signal noise”, causing stress signals to either be distorted in transmission or to be chronically activated, leaving the cells in a high-energy-consuming and inefficient “alert state” for a long time [71]. Under this continuous stress condition, cellular resources are continuously consumed for membrane repair and signal maintenance, thereby accelerating physiological exhaustion and systematically inhibiting high-energy-consuming secondary metabolic processes, including the biosynthesis of AFs [27].

## 5. Cell Wall Synthesis

The cell wall is the definitive interface for fungal morphology, structure, and interaction with the external environment. In the “internal disintegration” process, cell wall destabilization is not merely physical damage but the ultimate manifestation of the interplay between internal metabolic disorder, signaling dysregulation, and structural synthesis collapse, marking the fungus’s transition from an organized growth pattern towards a disordered pre-senescent state.

### 5.1. Imbalance in Cell Wall Synthesis and Repair

When exposed to external stress, fungi typically activate the cell wall integrity (CWI) pathway to initiate defensive cell wall reinforcement, manifested as an enhancement of cell wall-related synthesis and remodeling processes [81]. In Aspergillus, cell wall stress induced by Congo red or calcofluor white can cause apical growth arrest, hyphal swelling, and elevated chitin and glucan contents, indicating that fungi rapidly enter a defensive wall-thickening state under stress conditions [82]. Chitin synthases are important execution nodes in this repair system. For example, deletion of ChsA in *A. niger* reduces cell wall integrity, increases stress sensitivity, and disturbs chitin homeostasis, suggesting that the CHS system is essential for maintaining wall strength under adverse conditions [83].

However, this compensatory repair is highly dependent on sufficient energy and carbon resources. The synthesis of chitin and glucan requires activated sugar precursors such as UDP-N-acetylglucosamine and UDP-glucose, whose production depends on sugar uptake and central carbon metabolism [84,85]. Recent studies in *A. flavus* showed that disruption of sugar transport systems increases sensitivity to cell wall stressors and changes wall composition, with reduced glucan and galactomannan but compensatory chitin accumulation [86]. Therefore, when exogenous microorganisms compete for carbon sources and the cell internally redirects energy to basic survival, the synthesis rate of cell wall precursors is likely unable to sustain the high-intensity restoration.

Another key problem is the mismatch between wall synthesis and wall remodeling. A stronger cell wall does not simply depend on more polysaccharide deposition, but on the coordinated processes of synthesis, cross-linking, local hydrolysis, transport, and reassembly. In A. fumigatus, the EH domain protein EdeA participates in endocytosis, hyphal polarity, and cell wall integrity; its loss causes obvious wall defects, indicating that correct wall assembly requires membrane trafficking and localized remodeling [87]. Similarly, disruption of maiA does not markedly reduce total chitin, but decreases β-glucan content and causes sensitivity to Congo red and calcofluor white, together with abnormal wall architecture [88]. These results suggest that producing wall material is not equivalent to assembling a stable wall structure.

Thus, the initially thickened cell wall may represent compensatory but unstable reinforcement rather than true strengthening. Under prolonged stress, insufficient precursor supply, impaired membrane transport, ROS-mediated damage, and possible microbial enzymatic degradation may jointly weaken wall quality. Although chitin or glucan deposition may increase temporarily, the wall can become locally loose, unevenly assembled, and mechanically fragile. As stress continues, transcriptional and epigenetic regulation of wall homeostasis may also be disrupted; for example, loss of the histone acetyltransferase Sas3 in A. fumigatus leads to clear cell wall-related phenotypes [89]. In addition, nitrate assimilation has been shown to compensate for cell wall biosynthesis defects under specific conditions, further indicating that wall repair is tightly linked to metabolic reconfiguration [90]. Consequently, the fungal wall may gradually shift from defensive thickening to ineffective repair, with structural failure first appearing at mechanically vulnerable regions such as hyphal tips and branches.

### 5.2. Cytoskeletal Collapse and Loss of Polarized Growth

Polarized hyphal growth depends on the precise coordination of the apical growth machinery, including actin microfilaments, microtubules, vesicle trafficking systems, membrane fusion processes, and regulatory proteins [91,92]. During normal growth, wall precursors and membrane materials are continuously transported to the hyphal apex, where they are deposited in a spatially restricted manner to support tip extension. This process requires adequate ATP supply, an intact membrane system, and dynamic cytoskeletal organization. Therefore, when external stress disrupts energy metabolism, membrane integrity, or redox balance, the material and structural basis of polarized growth is directly weakened.

Under sustained stress, ROS accumulation and metabolic insufficiency may interfere with actin polymerization, microtubule stability, and vesicle transport. As a result, vesicles carrying wall components can no longer be efficiently delivered to the apex, and the Spitzenkörper-like apical organization becomes unstable. Once this transport system is disturbed, newly synthesized wall materials may be deposited in a disordered manner rather than concentrated at the growth tip. This explains why stressed hyphae often display apical swelling, irregular branching, and loss of directional extension. In this sense, cytoskeletal collapse and cell wall repair failure are not separate events, but mutually reinforcing processes: defective cytoskeletal transport impairs wall assembly, while weakened wall integrity further increases mechanical stress on the hyphal apex.

The regulatory core of polarized growth is also highly sensitive to stress signals. Rho-family GTPases, especially Cdc42 and RacA, control actin organization, vesicle targeting, hyphal morphology, and cell wall deposition. Recent studies in *A. flavus* showed that Cdc42 and RacA not only participate in morphogenesis, but also jointly regulate oxidative balance, energy metabolism, cell wall composition, and AF biosynthesis. Their absence causes significant morphological abnormalities, disturbed wall composition, and altered secondary metabolism [42]. This indicates that under continuous stress, disruption of the Rho GTPase network may simultaneously impair polarized growth, redox homeostasis, and toxin-related metabolic programs.

Upstream regulators of Rho GTPases further support this connection. GerA, a Rho guanine nucleotide exchange factor in A. flavus, has been shown to regulate growth, development, and AF biosynthesis [93]. This suggests that the polarity-regulating Rho signaling module is not only a morphogenetic pathway, but also a hub linking fungal development, stress adaptation, and secondary metabolism. Therefore, when exogenous stress persists, the fungus may experience a progressive failure of polarity control: energy shortage limits cytoskeletal dynamics, ROS damages membrane and protein systems, and stress signaling disturbs Rho GTPase-mediated spatial regulation. Eventually, the hyphae lose their ability to maintain stable apical extension, leading to swollen tips, abnormal branching, growth arrest, and collapse of polarized development.

## 6. Conclusions and Prospect

### 6.1. Conclusions

Microbial AF-control processes have traditionally been interpreted primarily as the direct chemical or enzymatic transformation of aflatoxin molecules by exogenous microorganisms or their catalytic products. However, accumulating evidence from transcriptomic, metabolomic, and cell biological studies suggests that, in microbial interaction systems containing viable toxigenic fungi, aflatoxin reduction may also involve complex physiological responses triggered by sustained microbial pressure. Based on these observations, this review proposes the conceptual framework of the internal disintegration effect, which describes how persistent microbial-induced stress may progressively affect signal transduction, transcriptional regulation, lipid and energy metabolism, cell wall integrity, and cellular morphology, ultimately reducing the toxin-producing capacity of toxigenic fungi.

Importantly, the internal disintegration framework should not be interpreted as a redefinition of aflatoxin biodegradation. Biodegradation should remain reserved for the direct chemical or enzymatic transformation of existing aflatoxin molecules. Instead, the proposed framework complements conventional biodegradation mechanisms by providing a physiological perspective on how microbial interactions may suppress aflatoxin accumulation through progressive remodeling of viable toxigenic fungi. Furthermore, this model should not be regarded as a universal explanation for all reported aflatoxin reduction phenomena. In systems involving purified enzymes, cell lysates, or cell-free preparations, aflatoxin reduction is more appropriately attributed to direct degradation processes rather than fungal physiological remodeling.

Therefore, the internal disintegration model is most relevant to microbial interaction systems involving living toxigenic fungi and should be regarded as complementary to, rather than replacing, conventional biodegradation mechanisms (Table 7). Future studies integrating multi-omics analyses, metabolic flux measurements, and targeted functional validation will be essential to determine whether sustained microbial stress can indeed drive irreversible physiological decline in toxigenic fungi and to identify common regulatory modules underlying long-term aflatoxin suppression. Such efforts may facilitate the development of next-generation dual-control strategies that simultaneously target both existing toxin molecules and the physiological processes responsible for toxin production.

### 6.2. Experimentally Testable Predictions of the Internal Disintegration Framework

To facilitate future validation, the internal disintegration framework generates several experimentally testable predictions. First, suppression of AF biosynthesis is expected to occur prior to substantial reductions in fungal growth, indicating that toxin-producing capacity may decline before loss of viability. Second, AF production and fungal biomass may become partially decoupled, such that viable fungi exhibit markedly reduced toxin synthesis. Third, intracellular ROS is predicted to function as a causal driver of physiological remodeling rather than merely a stress marker; therefore, manipulation of cellular redox status should alter the degree of toxin suppression. Fourth, transcriptional reprogramming involving stress-responsive signaling pathways and AF biosynthetic regulators should precede visible morphological abnormalities. Finally, reductions in AF production are expected to occur together with coordinated alterations in redox balance, energy metabolism, lipid metabolism, and cellular integrity, reflecting a system-wide deterioration of physiological homeostasis. Verification of these predictions would provide critical evidence supporting the internal disintegration framework as a distinct mechanism underlying long-term suppression of AF biosynthesis in microbial interaction systems.

### 6.3. Prospect

The fundamental mechanism of the “internal disintegration effect” is not specific to AFs; it involves foreign microbes inducing physiological remodeling through sustained stress, which suppresses secondary metabolism. When they come into contact with rival microorganisms, a number of toxic fungus (including *Fusarium*, *Penicillium*, and other *Aspergillus* species) also experience oxidative stress, signaling pathway activation, energy metabolism reallocation, and cellular structural instability. As a result, this model may be extended to other microbial AF control systems.

Other important mycotoxin contaminants in grains include ochratoxin A (OTA), deoxynivalenol (DON), and zearalenone (ZEN). However, these toxins originate from different fungal genera and should be considered separately. OTA is produced primarily by Aspergillus and Penicillium species, whereas DON and ZEN are predominantly produced by Fusarium species. Previous studies have shown that microbial antagonists can reduce OTA accumulation through nutrient competition, secretion of antifungal metabolites, and interference with fungal physiological processes [94,95]. Because oxidative stress responses and secondary metabolism are closely interconnected in OTA-producing fungi, persistent microbial pressure may also contribute to OTA suppression through physiological remodeling rather than solely through direct inhibition of fungal growth or toxin degradation.

In contrast, DON and ZEN biosynthesis are regulated by distinct metabolic and signaling networks in *Fusarium* species. Nevertheless, antagonistic bacteria and yeasts have been reported to effectively inhibit *Fusarium* growth and toxin production [96,97,98,99,100]. Within the internal disintegration framework proposed in this review, it is conceivable that persistent microbial pressure may drive *Fusarium* into a defense-priority state by continuously depleting carbon and nitrogen sources, generating oxidative stress, and disrupting energy metabolism. Such physiological remodeling could redirect cellular resources away from secondary metabolism and contribute to the downregulation of toxin biosynthesis-associated gene clusters. However, compared with aflatoxin-producing fungi, direct evidence supporting an internal disintegration process in *Fusarium* toxin-control systems remains limited and warrants further investigation.

To sum up, the “internal disintegration effect” offers a novel conceptual perspective on microbial AF control that extends beyond conventional detoxification and emphasizes the progressive loss of toxin-producing capacity in viable toxigenic fungi under persistent microbial-induced stress. Nevertheless, although increasing evidence supports individual components of this framework, important knowledge gaps remain regarding the causal linkage among microbial pressure, physiological remodeling, and long-term suppression of AF biosynthesis, and the complete mechanistic sequence has not yet been experimentally demonstrated within a single integrated system. Future studies should not only establish unified microbial–fungal interaction models that simultaneously monitor transcriptomic, metabolic, redox, and toxin-production dynamics, but also distinguish toxin degradation from adsorption, fungal growth inhibition, and suppression of de novo AF biosynthesis. Moreover, reductions in AFB1 levels alone should not be regarded as definitive evidence of detoxification, and comprehensive identification of degradation products together with rigorous toxicity assessments are required to verify safety. Further validation under microbial–fungal coculture systems and realistic food and feed matrices, as well as evaluation of matrix-dependent efficacy, microbial biosafety, impacts on product quality, scalability, and regulatory feasibility, will be essential for practical application. Such efforts will help determine whether internal disintegration represents a distinct biological phenomenon or an emergent consequence of interconnected stress-response mechanisms, ultimately guiding the development of precise, environmentally friendly, durable, and mechanism-based strategies for long-term AF control.

## Figures and Tables

**Table 1:** Comparison of major analytical methods for aflatoxin detection in cereals.

Method	Detection Principle	Typical LOD	Typical LOQ	Advantages	Limitations
ELISA	Antibody–antigen recognition	ng/kg–μg/kg	μg/kg	Rapid, inexpensive, suitable for screening	Cross-reactivity, limited multiplex capability
HPLC-FLD	Chromatographic separation with fluorescence detection	0.01–1 μg/kg	0.05–5 μg/kg	High sensitivity and accuracy	Requires derivatization and laboratory equipment
LC–MS/MS	Chromatography coupled with tandem MS	<0.01 μg/kg	<0.05 μg/kg	Excellent sensitivity and specificity; multi-mycotoxin analysis	High cost and technical complexity
Near-Infrared Spectroscopy (NIR)	Spectral fingerprint analysis	Typically indirect prediction models	Typically indirect prediction models	Rapid, non-destructive, suitable for online monitoring	Lower sensitivity; requires calibration models
FT-NIR	Fourier-transform NIR spectroscopy	Typically indirect prediction models	Typically indirect prediction models	Improved spectral resolution and robustness	Strong dependence on model quality
Raman Spectroscopy	Molecular vibrational fingerprinting	μg/kg range (model dependent)	μg/kg range (model dependent)	Minimal sample preparation; non-destructive	Fluorescence interference; lower reproducibility
SERS	Surface-enhanced Raman spectroscopy	ng/kg–μg/kg	ng/kg–μg/kg	Ultra-high sensitivity	Complex substrate preparation
Electrochemical Sensors	Signal generation through electrochemical reactions	ng/kg–μg/kg	ng/kg–μg/kg	Portable and rapid	Sensor stability and matrix interference
Aptamer-based Biosensors	Aptamer-target recognition	ng/kg	ng/kg	High selectivity and rapid detection	Aptamer stability and standardization issues

**Table 2:** Classification of mechanisms contributing to microbial aflatoxin control and the position of the internal disintegration framework.

Comparison Dimension	Direct Degradation	Adsorption/ Sequestration	Growth Inhibition	Biosynthesis Suppression	Internal Disintegration Model	References
Control Level	Toxin-targeting mechanisms	Toxin-targeting mechanisms	Fungus-targeting mechanisms	Fungus-targeting mechanisms	Fungus-targeting mechanisms	[44,45]
Primary target	AFs molecules	AFs molecules	Toxigenic fungi	Toxigenic fungi	Toxigenic fungi; their metabolic regulatory network	
Relationship to Biodegradation	Biodegradation itself	Not biodegradation	Not biodegradation	Not biodegradation	Not biodegradation	
Core mechanism	Enzymatic or chemical transformation of AFs into less toxic products	Binding of AFs to microbial cells, cell wall components, or adsorbent materials	Reduction in fungal growth, viability, or biomass	Downregulation of AF biosynthetic pathways and regulatory genes	Persistent microbial-induced stress triggers oxidative imbalance, transcriptional reprogramming, metabolic remodeling, and progressive deterioration of toxin-producing capacity	[44,45,46]
Key indicators	Identification of degradation products; structural modification of AFs	AF binding capacity; reversible or irreversible adsorption	Reduced colony growth, sporulation, or fungal biomass	Reduced expression of *AflR*, *AflS*, and biosynthetic genes; lower toxin production rate	ROS accumulation, activation of stress signaling pathways, transcriptional reprogramming, lipid and energy metabolic remodeling	[45,47]
Requirement for Viable Fungi	Not necessarily	Not necessarily	Yes	Yes	Yes	
Effect on AF production	Indirect or absent	Absent	Reduced due to decreased fungal growth	Directly reduced	Systematically reduced through physiological remodeling	[47,48]
Primary outcome	Detoxification	Toxin removal/sequestration	Reduced fungal proliferation	Reduced aflatoxin biosynthesis	Long-term reduction in aflatoxin accumulation through physiological suppression of toxin biosynthesis.	[48,49]
Relationship to the Internal Disintegration Model	Complementary mechanism	Complementary mechanism	Contributing process	Major downstream outcome	Central conceptual framework of this review	

**Note:** A reduction in measured aflatoxin concentration does not necessarily indicate aflatoxin biodegradation. Only direct degradation involves the biochemical transformation of existing aflatoxin molecules and therefore constitutes aflatoxin biodegradation. Adsorption/sequestration, fungal growth inhibition, biosynthesis suppression, and the proposed internal disintegration framework may all contribute to reduced aflatoxin accumulation, but they should be considered distinct mechanisms of microbial aflatoxin control rather than biodegradation itself. The internal disintegration framework specifically describes stress-mediated physiological remodeling occurring in viable toxigenic fungi under persistent microbial pressure and therefore does not apply to purified enzyme systems, cell-free extracts, or isolated catalytic degradation systems.

**Table 3:** Comparison between conventional microbial antagonism and the internal disintegration effect.

Feature	Conventional Antagonism	Internal Disintegration
Primary target	Fungal growth	Toxin-producing physiology
Main outcome	Reduced biomass	Reduced toxigenic capacity
ROS role	Secondary response	Central integrative driver
Requirement for growth inhibition	Usually required	Not necessarily required
Reversibility	Often reversible	Progressive physiological collapse
Key indicators	Biomass, colony diameter	ROS, transcriptome, metabolome, polarity defects
Typical endpoint	Growth suppression	Loss of toxin biosynthetic competence

**Table 4:** Summary of the roles of bZIP transcription factors in oxidative stress response and aflatoxin biosynthesis in *A. flavus*.

Regulatory Category	Representative bZIP Factors	Effect on AFB_1_ Production	Possible Regulatory Implication
Stress-responsive and context-dependent regulators	AtfA, AtfB	Deletion significantly reduced AFB_1_ production and altered *AflR/AflS* expression	Coordinate oxidative stress adaptation and secondary metabolism; regulatory effects may vary with stress intensity, developmental stage, and physiological conditions
Positive contributors to AF biosynthesis	Atf2, Atf3, Atf4, Atf5, MeaB	Deletion reduced AFB_1_ production to varying degrees	Participate in stress-mediated regulation of aflatoxin biosynthesis
Metabolism-linked regulators	CpcA, MetR	Deletion reduced AF production and affected amino acid or sulfur metabolism	Connect nutrient metabolism with secondary metabolism
Potential negative regulators	JlbA	Deletion increased AFB_1_ production	May repress AF biosynthesis under certain environmental conditions
Weak or condition-dependent regulators	HapX, Zip1, Zip6, ZipP	Minor or variable effects on AFB_1_ production	Function may depend on specific environmental or stress conditions

**Table 5:** A summary of the regulatory roles of CreA in carbon metabolism, fungal development, and aflatoxin biosynthesis in *A. flavus*.

Regulatory Level	Phenotype Observed in ΔCreA	Biological Implication
Vegetative growth	Reduced colony growth under different carbon sources	CreA supports normal carbon-dependent growth
Asexual development	Conidial production markedly decreased	CreA is required for sporulation and reproductive development
Aerial hyphae/surface property	Aerial hyphae decreased and hydrophobicity weakened	CreA affects colony architecture and surface development
Carbon utilization	α-amylase activity increased and was no longer repressed by glucose/sucrose/maltose	CreA mediates carbon catabolite repression of hydrolytic enzymes
Sclerotial formation	Sclerotia increased under dark and light conditions	Loss of CreA may shift metabolism toward stress/dormancy structures
AF biosynthesis	AFB_1_ production sharply reduced	CreA positively contributes to aflatoxin biosynthesis
AF gene expression	aflR, aflD, aflM, aflO, aflP downregulated	CreA regulates AFs partly through the canonical aflatoxin gene cluster

**Table 6:** Temperature-associated lipid metabolic characteristics and aflatoxin production in *Aspergillus flavus*.

Culture Temperature	28 °C (AFB1-Permissive Condition)	37 °C (AFB1-Suppressive Condition)
AFB1 production	Higher	Lower
Polyunsaturated fatty acids (C18:2, C18:3)	Higher abundance	Lower abundance
Degree of lipid unsaturation	Higher	Lower
Triglycerides containing unsaturated acyl chains	More abundant	Less abundant
Fatty acid metabolic gene expression	Baseline/reference level	Altered expression profile
Oxidative stress response	Lower oxidative stress indicators reported	Higher oxidative stress indicators reported
Overall lipid profile	Enriched in unsaturated lipid species	Shift toward more saturated lipid species

**Table 7:** Scope and Boundary Conditions of the Internal Disintegration Model.

Scenario	Included?
Living fungal–microbial coculture	yes
Long-term microbial stress	yes
Oxidative stress–induced physiological remodeling	yes
Pure toxin adsorption	no
Purified enzyme degradation	no
Cell-free degradation systems	no
Instant fungicidal killing	no
Temporary nutrient limitation	Partial

## Data Availability

No new data were created or analyzed in this study. Data sharing is not applicable to this article.
